# Adenocarcinoma of the Lung Presenting with Intrapulmonary Miliary Metastasis

**DOI:** 10.7759/cureus.5430

**Published:** 2019-08-19

**Authors:** Saran Pillai, Adnan Khan, Sana Khan

**Affiliations:** 1 Emergency Medicine, Kerala Institute of Medical Sciences Hospital, Trivandrum, IND; 2 Critical Care, Freeman Health System, Joplin, USA; 3 Internal Medicine, Sindh Medical College, Karachi, PAK

**Keywords:** adenocarcinoma, miliary, metastasis, metastatic lung adenocarcinoma, mimic, tb mimic, miliary tuberculosis

## Abstract

Miliary mottling on imaging is usually infectious in etiology and is less commonly seen with metastatic cancers. The cancers that are reported to cause miliary metastases include secondaries from cancers of the thyroid, kidney, trophoblasts, etc. Here, we report a case of a 63-year-old female who presented with prolonged cough and shortness of breath and whose imaging showed diffuse bilateral miliary nodules. Bronchoscopy with a transbronchial biopsy confirmed the diagnosis as adenocarcinoma of the lung with intrapulmonary miliary metastasis. Treatment with a combination of pemetrexed and carboplatin was not helpful and cancer had spread diffusely across the lung on repeat imaging after three months. It is essential to consider this clinical presentation as a separate subtype, with specific treatment protocols as compared to primary adenocarcinoma of the lung.

## Introduction

Miliary mottling consists of numerous pulmonary opacities with a size of less than 3 mm scattered throughout the lungs on chest radiography [1]. The differential diagnosis is broad, including tuberculosis, fungal infections, occupational lung diseases, sarcoidosis, and metastatic disease among the most common causes [2]. The most common metastatic cancers leading to miliary metastasis are hematogenous metastasis from thyroid carcinoma, renal cell carcinoma, melanoma, osteosarcoma, colorectal carcinoma, testicular tumors, and, very rarely, seen with lung cancers [2-4]. We present a case of a 63-year-old female with lung adenocarcinoma who presented with intrapulmonary miliary metastasis.

## Case presentation

A 63-year-old woman presented to the clinic with a dry cough and shortness of breath for three weeks. A review of systems showed progressive fatigue, intermittent low-grade fevers with temperatures up to 100°F, and an unexplained 12-pound weight loss, all over the last three months. An esophagogastroduodenoscopy done a week back for the evaluation of her cough was unremarkable.

Past medical history was significant for recurrent pneumonia and negative for tuberculosis (TB). There was no family history, history of close contact with tuberculosis, or travel or incarceration history, though she worked as a nurse at an Alzheimer’s patient care facility. She had a 10 pack-year smoking history.

**Figure 1:**
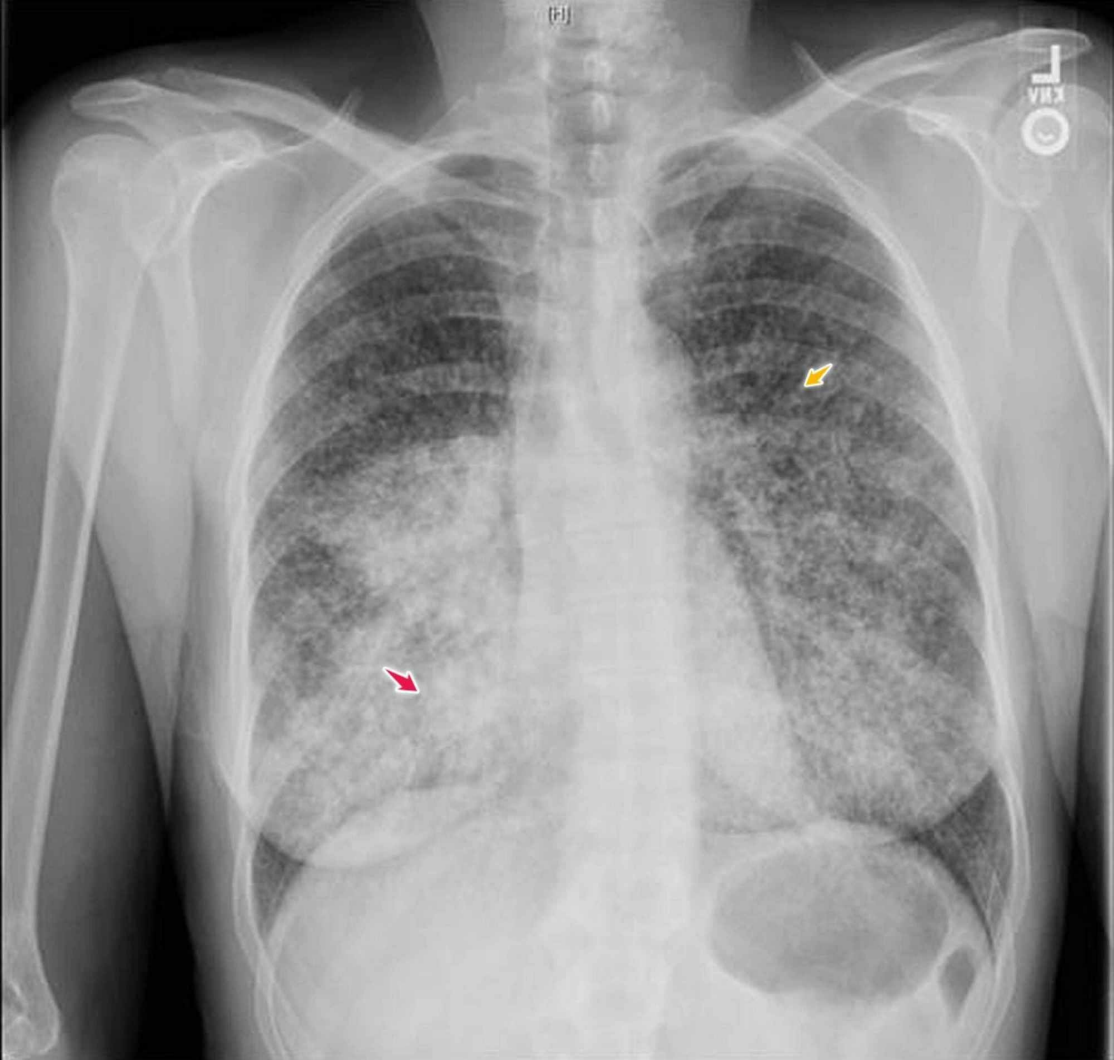
Chest X-ray, posteroanterior view Chest X-ray, posteroanterior view, showing extensive bilateral pulmonary infiltrates with a miliary pattern (orange arrow) and consolidation in the right lower lung field (pink arrow)

**Figure 2:**
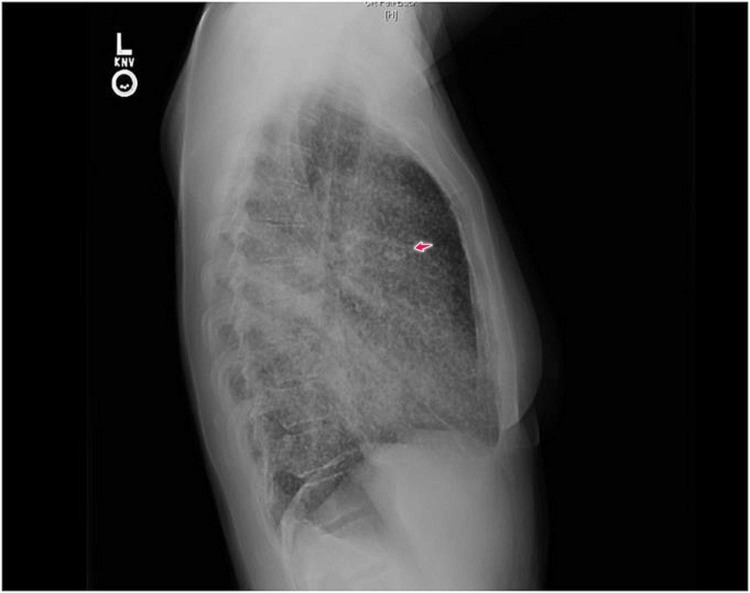
Chest X-ray, lateral view Chest X-ray, lateral view, showing extensive pulmonary infiltrates with a miliary pattern (arrow)

**Figure 3:**
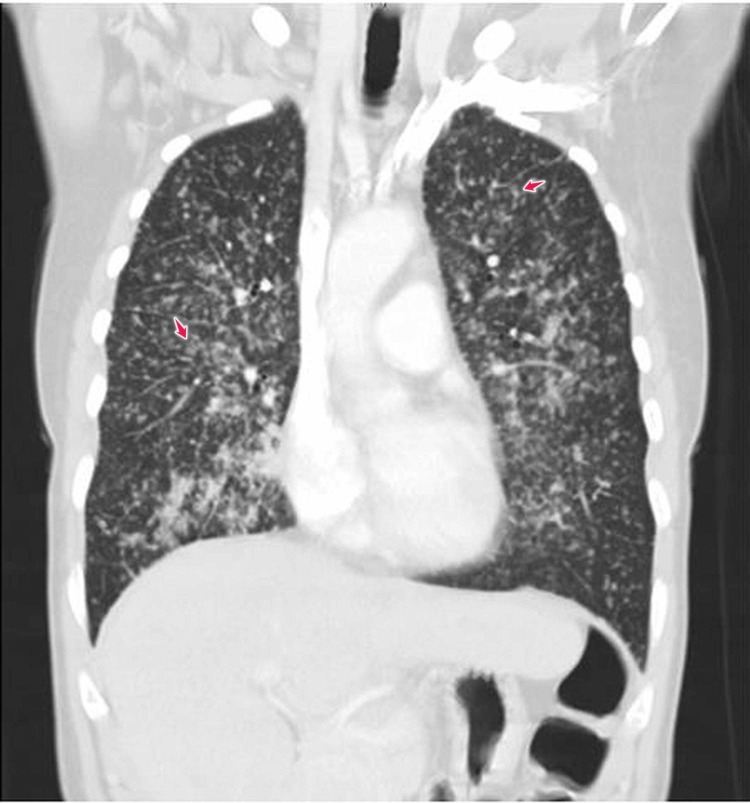
CT scan chest Chest computerized tomography (CT) scan with multiple bilateral miliary nodular infiltrates (arrows)

**Figure 4:**
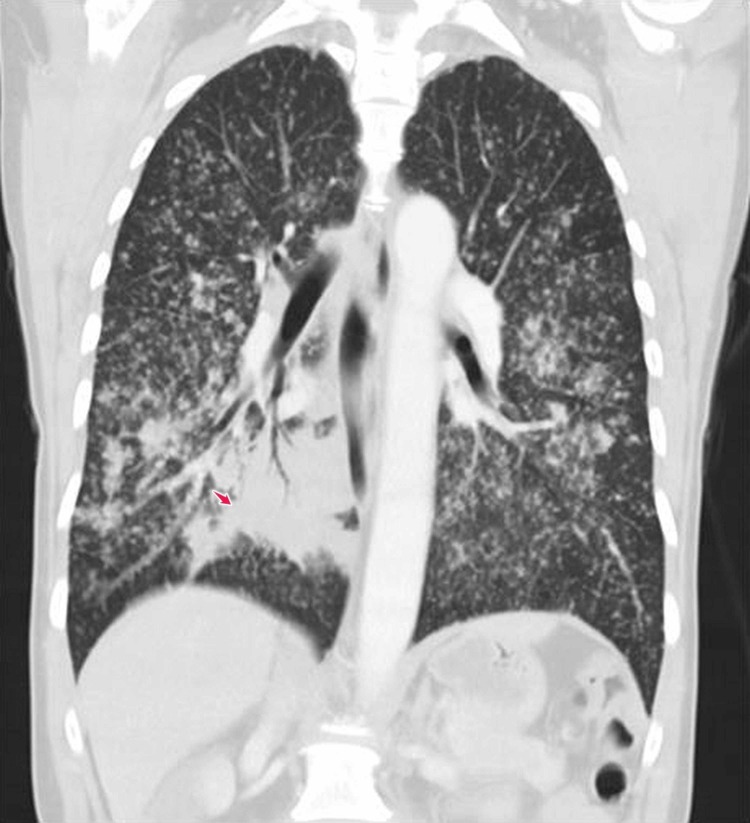
CT scan chest Computerized tomography (CT) scan of chest with a mass-like prominence in the right lower lung field (arrow)

Vital signs, physical examination, and laboratory testing were primarily benign, except for a respiratory exam that showed bronchial breathing 2 cm above the lung base in the right mid-scapular line. A chest X-ray showed extensive bilateral pulmonary infiltrates with a miliary pattern, and consolidation in the right lower lung field (Figures 1-2). Computed tomography (CT) scan of the chest confirmed multiple miliary nodular infiltrates throughout both the lungs and a mass-like prominence in the right infrahilar and right lower lung field with hilar and mediastinal lymphadenopathy (Figures 3-4). No other metastases were found on brain magnetic resonance imaging (MRI), abdominal CT, or pelvic CT imaging.

The patient was initially placed on airborne precautions. The following tests done to narrow down the differential were all negative: fungal serology and urine antigen testing for blastomycosis and histoplasmosis, quantiferon tuberculosis (TB) gold test, sputum acid-fast bacilli (three samples), tuberculin skin test, human immunodeficiency virus (HIV) antibody test, and hypersensitivity pneumonitis screen.

Fiberoptic flexible video bronchoscopy was performed and was macroscopically normal. Bronchioalveolar lavage (BAL), bronchial brushings, and fluoroscopy-guided transbronchial biopsies of the lung lower lobes were done. Gram stain and culture of BAL showed no organisms. Histological and cytological analysis of BAL, as well as a lung biopsy, showed an adenocarcinoma with a proliferation of glandular structures in a micropapillary configuration. Immunohistochemical analysis revealed the tumor cells as positive for thyroid transcription factor (TTF-1), napsin, wild type anaplastic lymphoma kinase (ALK), ROS1, and wild type epidermal growth factor receptor (EGFR).

She was started on a combination chemotherapy regimen of pemetrexed and carboplatin and underwent two cycles of chemotherapy within three months. Her treatment course was complicated by severe pancytopenia, neutropenic fever, and pulmonary emboli, which were managed with inpatient intensive care treatment with rivaroxaban, broad-spectrum antibiotics, blood products, and supportive care. Unfortunately, repeat imaging after three months showed the extensive progression of the miliary nodules as compared to the previous images. The patient declined further cycles of chemotherapy or alternate regimens and opted to follow supportive treatment. She was discharged to palliative care.

## Discussion

Lung carcinoma or bronchogenic carcinoma is a malignant neoplasm of the lung arising from the respiratory epithelium of the bronchus or bronchiole. It is the leading cause of cancer-related mortality, accounting for 90% of lung cancer-related deaths [5]. Lung cancer presents with predominantly respiratory symptoms as well as B symptoms and symptoms related to the obstruction of the airway or adjacent structures [6].

The tumor begins as a focus of atypical cells in the bronchial mucosa, which may progressively grow either into the bronchial lumen or into the bronchial wall or the adjacent lung parenchyma before it metastasizes into the lymph nodes and the distant organs [7]. Lung cancers can be broadly classified as small cell carcinomas and non-small cell carcinomas, the latter being subdivided into adenocarcinoma, squamous cell carcinoma, large cell carcinoma, and bronchial carcinoid tumors [8]. Adenocarcinoma and large cell carcinoma are usually peripheral in location, with pleural involvement, while most squamous and small cell carcinoma presents as a central mass with endobronchial growth [8].

Adenocarcinoma is the most common primary lung cancer accounting for 50% of cases and the most common lung cancer among nonsmokers and among women [6]. It is composed of malignant glandular epithelium with varying degrees of differentiation and architecture of neoplastic glands. Adenocarcinoma may demonstrate an acinar, papillary, micropapillary, lepidic, or solid growth pattern, with either mucin or pneumocyte marker expression [9].

**Figure 5:**
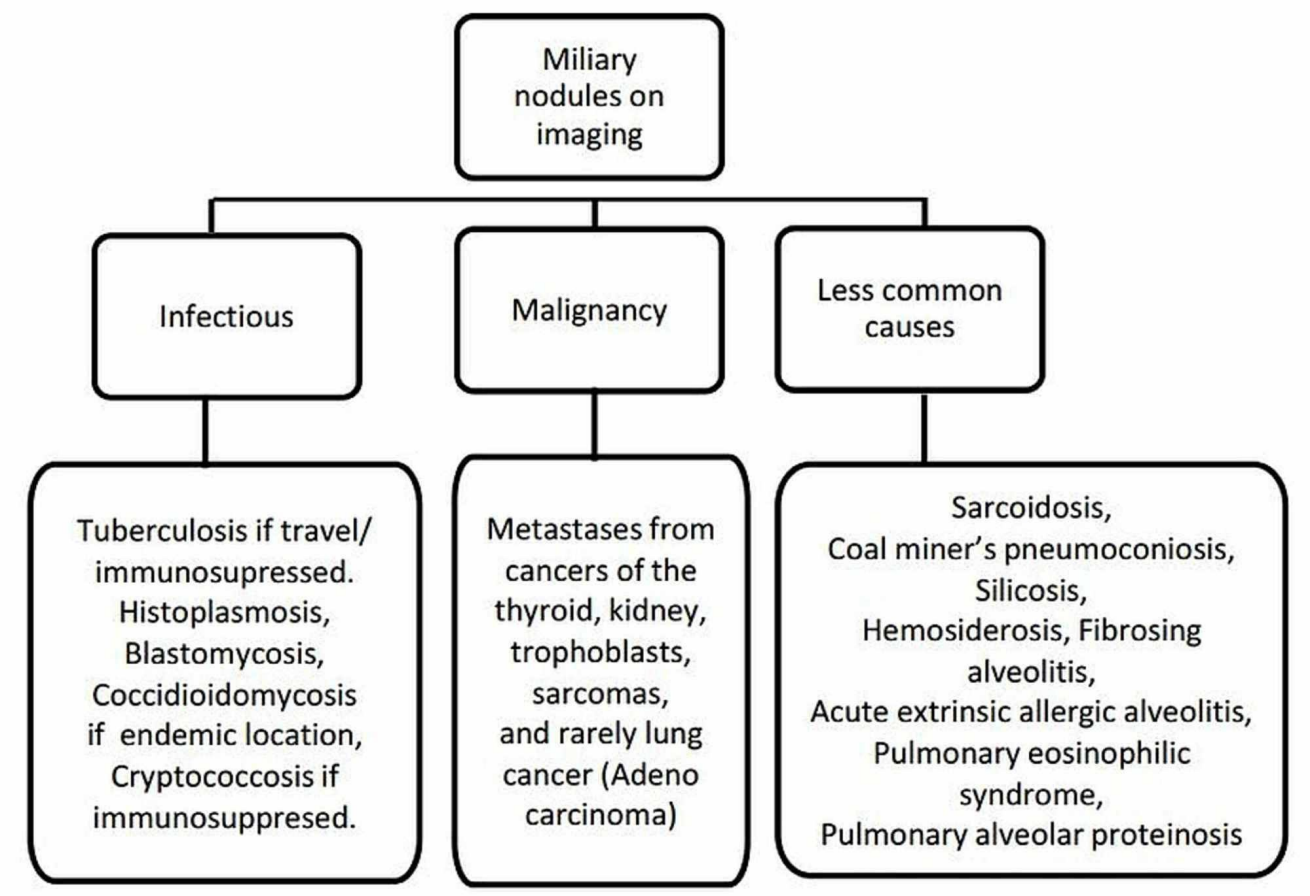
Differential diagnosis of miliary nodules on imaging

Miliary micronodules seen in a CT scan may be classified based on their distribution into the centrilobular, perilymphatic, and random patterns seen commonly in infectious bronchiolitis, sarcoidosis, and hematogenous metastases, respectively (Figure 5) [1]. A random pattern, as seen in our patient, consists of micronodules randomly scattered along the secondary lobules with symmetric involvement of the basal part of the lungs [1].

The miliary pattern in infections and metastases occurs when organisms or tumor cells spread through systemic vasculature, get lodged in the capillary beds and then proliferate locally. In adenocarcinoma of the lung with intrapulmonary miliary metastasis, the underlying mechanism is unclear, though it is possible that the masses were dispersed through the pulmonary vasculature [10]. Unlike miliary tuberculosis where the micronodules range in size from 1 mm to 3 mm, miliary metastatic lesions may vary in size up to 1 cm [11].

An extensive review of the literature showed 15 cases of adenocarcinoma of the lung presenting with intrapulmonary miliary metastasis reported from two case series [12-13] and five case reports [1-2,10-11]. While adenocarcinomas of the lung are commonly associated with mutations in EGFR, KRAS, and ALK, adenocarcinomas that present with intrapulmonary miliary metastasis seem to be associated with EGFR mutations alone (especially exon 19 deletion and exon 21 point mutation) [1,14-16]. Furthermore, adenocarcinomas that have both a miliary pattern and an EGFR mutation seem to have shorter survival times, probably due to the increased tumor burden in this case [1]. They also seem to have improved response to EGFR-tyrosine kinase inhibitors (TKI) [14]. Similarities among the cases reported in literature included the clinical profile, poor prognosis, and unfavorable treatment response with non-TKI chemotherapy. This seems to suggest that this presentation should be identified as a separate subtype with unique treatment protocols with EGFR-TKI [10]. Unfortunately, the patient in our case opted to discontinue chemotherapy after three cycles due to side effects, before a new chemo regimen with TKI could be started.

## Conclusions

The most common causes of miliary mottling on lung imaging include infectious causes like TB and secondaries from malignant tumors of the thyroid, kidney, trophoblasts, etc. Though rare, primary adenocarcinomas of the lung can also have intrapulmonary miliary metastasis, in which case, it has an association with EGFR mutations, has a poorer prognosis, and has an unfavorable treatment response to non-TKI chemo regimens. It is essential to consider this presentation as a separate subtype with unique treatment protocols when compared to primary adenocarcinoma of the lung.
